# Properties, Microstructure Development and Life Cycle Assessment of Alkali-Activated Materials Containing Steel Slag under Different Alkali Equivalents

**DOI:** 10.3390/ma17010048

**Published:** 2023-12-22

**Authors:** Xin Ji, Xiaofeng Wang, Xin Zhao, Zhenjun Wang, Haibao Zhang, Jianfei Liu

**Affiliations:** 1 School of Materials Science and Engineering, Chang’an University, Xi’an 710061, China; xinji@chd.edu.cn (X.J.); haibaozhang@chd.edu.cn (H.Z.); jianfeiliu@chd.edu.cn (J.L.); 2 Henan Provincial Communications Planning & Design Institute, Zhengzhou 450052, China; 3 Shaanxi Provincial Communications Planning & Design Institute, Xi’an 710065, China; xinzhao710065@163.com; 4 Xi’an Key Laboratory of Modern Transportation Function Materials, Xi’an 710064, China

**Keywords:** steel slag, alkali equivalent, alkali-activated materials, properties, microstructure, environmentally friendly

## Abstract

To improve solid waste resource utilization and environmental sustainability, an alkali-activated material (AAM) was prepared using steel slag (SS), fly ash, blast furnace slag and alkali activators in this work. The evolutions of SS content (10–50%) and alkali equivalent (4.0–8.0%) on workability, mechanical strength and environmental indicators of the AAM were investigated. Furthermore, scanning electron microscopy, X-ray diffraction and nuclear magnetic resonance techniques were adopted to characterize micromorphology, reaction products and pore structure, and the reaction mechanism was summarized. Results showed that the paste fluidity and setting time gradually increased with the increase in SS content. The highest compressive strength was obtained for the paste at 8.0% alkali equivalent due to the improved reaction rate and process, but it also increased the risk of cracking. However, SS was able to exert a microaggregate filling effect, where SS particles filling the pores increased the structural compactness and hindered crack development. Based on the optimal compressive strength, global warming, abiotic resource depletion, acidification and eutrophication potential of the paste are reduced by 76.7%, 53.0%, 51.6%, and 48.9%, respectively, compared with cement. This work is beneficial to further improve the utilization of solid waste resources and expand the application of environmentally friendly AAMs in the field of construction engineering.

## 1. Introduction

Related research has shown that an alkali-activated material (AAM) composed of aluminosilicate powder and alkali metal solution seems to be an excellent alternative to ordinary Portland cement (OPC) [1]. Most industrial solid wastes, such as metakaolin, fly ash (FA) and ground granulated blast-furnace slag (GGBS), can be reused as solid precursors to prepare AAMs [2]. Commonly used alkali activators include hydroxides, carbonates, sulfates and silicates, among which the most popular are Na_2_SiO_3_ and NaOH [3]. Compared with OPC, AAMs have the advantages of low carbon dioxide emission and low energy consumption because they do not need to be calcined [4]. Moreover, AAMs also exhibit excellent properties such as early strength, permeability, high temperature resistance and acid corrosion [5,6,7,8].

At present, industrial solid waste not only has a low utilization rate but also occupies a lot of land, causing regional environmental degradation [9]. However, most solid wastes are rich in silica and aluminum oxide with potential gelation activity, which can be used as solid precursors to produce AAMs. Herein, FA and GGBS are widely used as cementitious admixtures due to their abundant reserves, low cost and stable nature [10]. During alkali activation, high-calcium FA is more inclined to form calcium-aluminosilicate-hydrate, which is similar in structure to tobermorite mineral; while low-calcium FA is alkali-aluminosilicate-hydrate, in which tetrahedral units of silicate and aluminate are connected to form a three-dimensional reticular spatial structure, and cations such as alkali metals and calcium ions compensate for the charge balance associated with the substitution of aluminum ions [11].

However, it is not advised to utilize alkali-activated FA as a building material due to its demonstrated low compressive strength and cementitious capabilities [12,13]. To overcome these shortcomings, the properties of activated FA are frequently enhanced by adding GGBS. Adding GGBS into the FA matrix not only improves the reaction rate and compressive strength but also promotes dense microstructure formation. However, the incorporation of GGBS can also cause rapid coagulation and large shrinkage of the paste, which not only increases the difficulty of construction but also increases the risk of matrix cracking [14,15]. For this reason, researchers usually add superabsorbent polymers, blended cement and fibers to solve these problems [16,17,18]. However, these materials are not only expensive but also have high carbon emissions. Therefore, research on other industrial solid wastes with potential utilization value is necessary.

Steel slag (SS), a by-product of the steelmaking process, accounts for about 15–20% of steel output [19]. Due to the low utilization rate of SS, most SS is discarded indiscriminately. However, SS is rich in CaO, SiO_2_ and Al_2_O_3_, which endow SS with potential gelation or pozzolanic activity. The grinding of SS, GGBS and cement clinker to produce blended cement in a non-calcined condition is a green and energy-efficient way of production [20]. However, relevant studies have confirmed that SS can inhibit cement hydration, which limits the improvement of mechanical properties and durability of the paste [21,22]. Zhao et al. [23] used the synergistic hydration effect between SS and GGBS to prepare a mortar with a compressive strength of 25.8 MPa at 28 days; they found that SS increased the pH of early pore solution and conductivity, which accelerated the dissolution of the glassy phase of the GGBS and volcanic ash reaction. Song et al. [24] activated SS and high-calcium FA using Na_2_SiO_3_-NaOH, and they found that the compressive strength of the paste at 28 days was the highest (25.48 MPa) when the substitution rate of SS was 20%. Furthermore, Song [25] incorporated SS-GGBS into alkali-activated high-calcium FA and found that SS-GGBS could improve the workability and mechanical properties, and the capillary pores in the paste were gradually refined into gel pores.

In summary, using SS as an auxiliary cementing material is a current research hotspot. However, the compressive strength of the paste is still low in the absence of cement or in the presence of a large SS content, which limits the expansion of its application in emergency-repair engineering such as road repair and grouting reinforcement. In addition, the effect of alkali content on the properties of FA-GGBS systems containing different SS contents has not been reported yet. Therefore, it is necessary to clarify the effects of alkali content on the properties and microscopic mechanism of the SS-FA-GGBS system.

In this work, the effects of alkali equivalent on the properties and microstructure of the AAM containing SS were investigated. The fluidity, setting time, mechanical properties and environmental benefits of the paste under different alkali equivalents and SS contents were analyzed. Furthermore, based on the microstructure characterization, the mechanism of the effect of SS contents and alkali equivalents on the AAM in the ternary system was explored. This work is of great significance for expanding the application of SS in the field of construction engineering.

## 2. Materials and Methods

### 2.1. Materials

**Figure 1:**
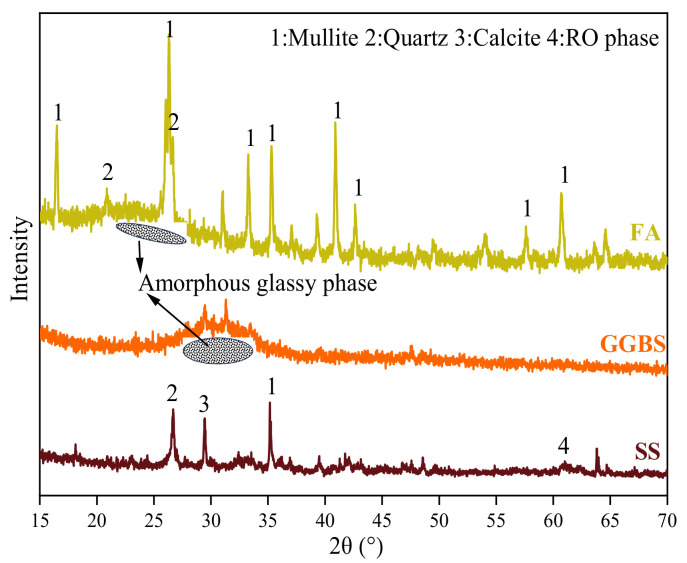
Mineral phases of solid wastes.

**Figure 2:**
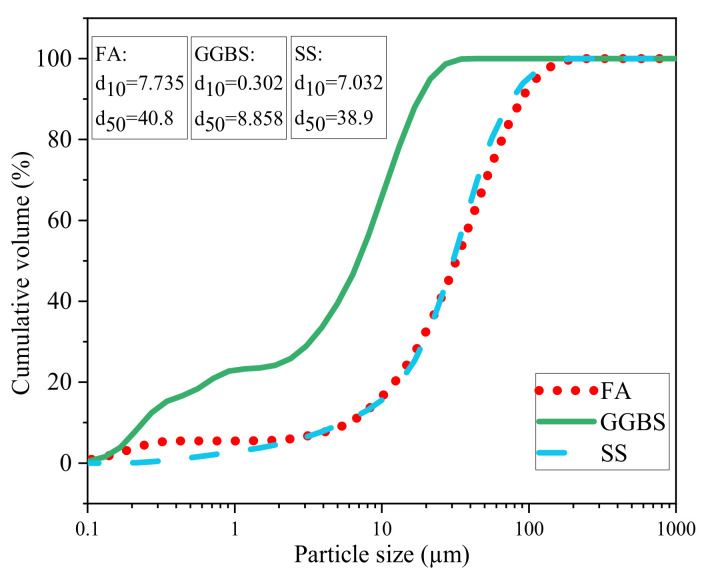
Particle size distribution of solid wastes.

FA, SS and GGBS were used as the main calcium aluminosilicate source materials. Chemical composition and mineral composition of the raw materials were determined using an X-ray fluorescence spectrometer-1800ASF(E) (Shimadzu, Japan) (XRF) and AXS D8-Advance X-ray diffraction (Bruker, Germany) (XRD), as shown in Table 1 and Figure 1. As can be seen, the order of the amorphous glassy phase content in the raw materials is GGBS > FA > SS, which indicates that GGBS contains more reactive silica. Their size distributions were determined using a particle analyzer, as shown in Figure 2. Furthermore, the autoclaved stability of the SS used was tested to ensure the volume stability of SS, and the result was 0.34%, which met the requirements of ASTM C151-09 [26] (0.5% expansion ratio for cement). The alkali activator used was composed of Na_2_SiO_3_ solution and NaOH (purity is 96%), and its chemical and physical properties are shown in Table 2.

### 2.2. Mix Design

Twelve pastes were synthesized in this work, and the mix proportions are shown in Table 3. The fixed content of FA in the AAM is 30%, and SS and GGBS were used as mineral additives constituting a total of 70%. In particular, GGBS was gradually replaced by SS, with SS accounting for a total of 10 to 50%. The modulus (SiO_2_/Na_2_O molar ratio) of the modified Na_2_SiO_3_ solution used in this work is 1.6, which can be obtained by adding NaOH to Na_2_SiO_3_ solution (see Equation (1)). Based on the Na_2_O equivalent (N_e_) in the modified Na_2_SiO_3_ with 1.6 modulus (see Equation (2)), the AAM was mixed with 4.0, 6.0 and 8.0 wt% N_e_ of the modified Na_2_SiO_3_ by the external addition method. In addition, the water–binder ratio was designed as 0.45. Here, the water in the alkali activator was adopted as part of the total water consumption.

(1) mNaOH=100(ϖSiO2+ϖNa2O)MNa2O+3.2MSiO2×2α−2ββ×MNaOHPNaOH

(2) $$m_{Na_{2}O}=\frac{100(\varpi_{SiO_{2}}+\varpi_{Na_{2}O})}{M_{Na_{2}O}+3.2M_{SiO_{2}}}\times (1+\frac{\alpha -\beta }{\beta })\times M_{Na_{2}O}$$

where α and β are the initial modulus of the Na_2_SiO_3_ solution and the modulus of the modified Na_2_SiO_3_ solution, respectively; $m_{NaOH}$ is the NaOH mass added to 100 g of the modified Na_2_SiO_3_ solution in g; $m_{Na_{2}O}$ is the Na_2_O mass in 100 g of the modified Na_2_SiO_3_ solution in g; $\varpi_{Na_{2}O}$ and $\varpi_{SiO_{2}}$ are the mass ratios of Na_2_O and SiO_2_ in the initial Na_2_SiO_3_ solution, respectively in %; $M_{Na_{2}O}$, $M_{SiO_{2}}$ and $M_{NaOH}$ are the molar masses of Na_2_O, SiO_2_ and NaOH, respectively, in g/mol; $P_{NaOH}$ is the purity of NaOH.

### 2.3. Specimen Preparation

Based on the mix proportions, the SS, GGBS and FA powders were first mixed in a mixer for 300 s for even dispersion. Then, we added the required water and activator and continued mixing for 180 s to get a fresh paste. The fresh pastes were poured into a 40 × 40 × 160 mm^3^ triplex metal molds and cured at ambient temperature for 24 h and then demolded and cured in a curing room (20 ± 2 °C, 95% RH) for 3, 7 and 28 days.

### 2.4. Characterization Methods

#### 2.4.1. Workability and Mechanical Properties of AAM

The setting time and fluidity of the paste were tested according to the Chinese standard GB/T 1346-2011 [27] and Chinese standard GB/T 8077-2012 [28], respectively. In reference to the Chinese standard GB/T 17671-1999 [29], a universal testing machine was used to test the mechanical properties of the samples cured for 3, 7 and 28 days. Each group of samples was tested three times, and the average value was taken as the final result.

#### 2.4.2. Microstructure Characterizations

XRD was conducted to analyze the mineral composition in the hydration products. After strength testing, small pieces of broken samples were crushed with an agate mortar and affixed to glass slides. Then, they were placed together in a diffractometer with a CuKa source at 40 kV and 40 mA. Samples were scanned at 10–70° (2θ) at a rate of 30 s/°. Similarly, crushed samples were selected to investigate the microstructure using S-4800 scanning electron microscopy (Hitachi, Tokyo, Japan) (SEM) under 10 kV accelerating voltage and a work distance of about 15 mm. The test samples were fixed to the sample holder with a conductive adhesive and sprayed with gold before testing. In addition, the pore structure was tested using MacroMR 12-150H-I nuclear magnetic resonance (Niumag, Suzhou, China) (NMR). Before testing, the samples’ interiors were filled with water using a Neld-CCM vacuum water saturation machine (Nel-Der, Changshu, China) for 3 h. Then, the sample was put into the MRI machine for testing. The magnetic field strength was 0.3 T, the magnet temperature was 32 °C and the pulse frequency range was 1 to 30 Hz.

#### 2.4.3. Life Cycle Assessment

The potential environmental impacts of the materials and mixtures were evaluated using life cycle assessment (LCA). The LCA procedure was standardized in the ISO 14040 [30] series and was performed in a CML 2016 method. Among them, the OpenLCA 2.0 software and Ecoinvent 3.9.1 database were used. Global warming potential (GWP), abiotic resource depletion potential (ADP), acidification potential (AP) and eutrophication potential (EP) were selected as the environmental indicators, and their results were calculated with 1 m^3^ as the functional unit.

## 3. Results and Discussion

### 3.1. Workability

#### 3.1.1. Effects of N_e_ and SS on Setting Time of AAM

**Figure 3:**
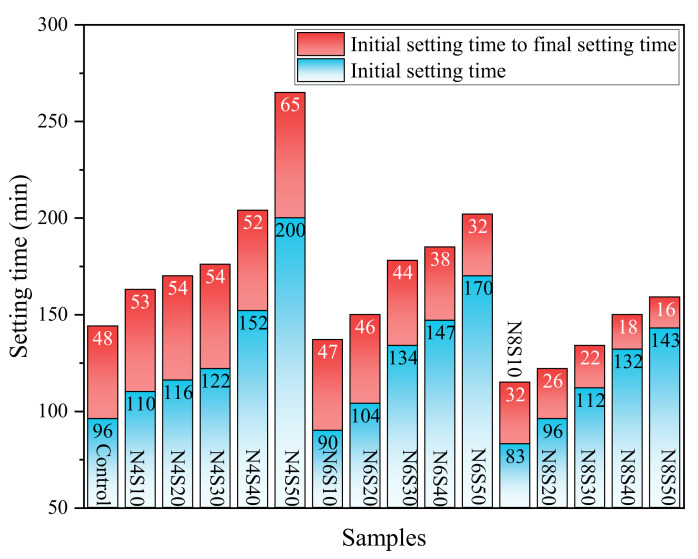
The setting time of AAM at different SS contents and N_e_.

Setting time is an important parameter, which determines the pumping distance. Figure 3 shows the setting time results of the AAM. As can be seen, the initial setting time of the AAM ranges from 83 to 200 min, and the final setting time ranges from 115 to 265 min. As the SS content increases, the setting time of the paste increases significantly. At the same N_e_ level, the initial setting times of N4S50, N6S50 and N8S50 increased by 81.8%, 88.9% and 72.3%, respectively, compared with the sample mixed with 10% SS; the final setting times increased by 62.6%, 47.4% and 38.3%, respectively. Since the vitreous content in GGBS is much greater than that of SS, the increase in the content of SS makes the gelation activity of the paste worse, increasing the setting time. The paste setting time gradually shortens as the N_e_ level increases. Compared with 4.0% N_e_, the average setting time of the samples mixed with 6.0% and 8.0% N_e_ reduced by 12.9% and 30.5%, respectively. This is because the increase in alkali concentration gradually accelerates the leaching of calcium, aluminum and silicon, promotes the reaction process [31] and thereby shortens the setting time.

#### 3.1.2. Effects of N_e_ and SS on Fluidity of AAM

**Figure 4:**
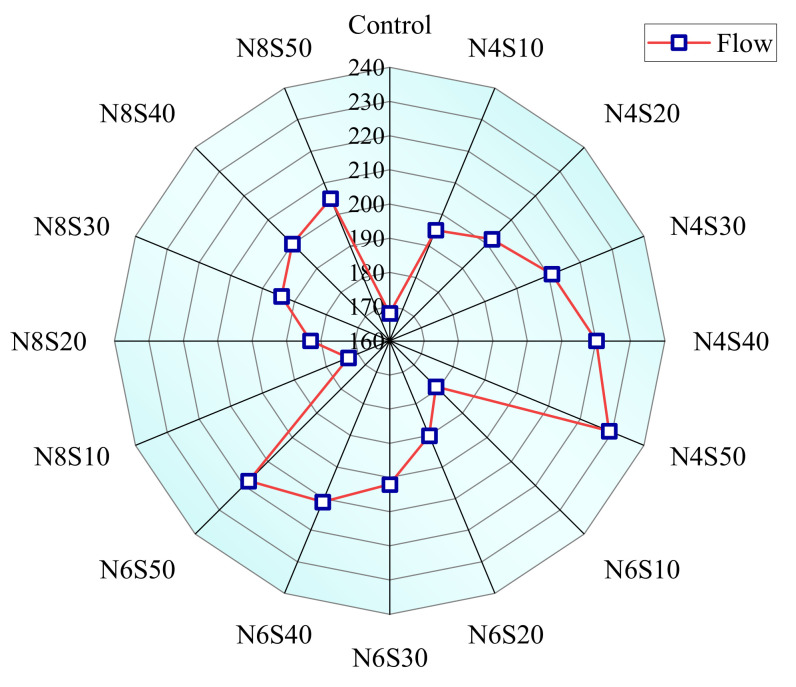
Fluidity of AAM at different SS contents and N_e_.

The flow of the AAM is shown in Figure 4. The flow of the control group is 168 mm. As can be seen, AAM with 4.0% N_e_ has the greatest flow. The flow of the AAM increased from 195 to 229 mm as the SS content increased from 10 to 50%. When N_e_ is 8.0%, the AAM has the least liquidity, and its flow range is 173 to 205 mm. Compared to the pastes with 8.0% N_e_, the average flow of the pastes with 4.0% N_e_ increased by 4.71% and the pastes with 6.0% N_e_ increased by 10.68%. The increase in N_e_ can speed up hydration and reduce the remaining free water in the paste; thus, the flow is reduced. Furthermore, the fluidity of the paste increases gradually as the SS content increases when N_e_ is constant. Usually, the apparent density of SS is higher, and in the case of mixing AAM by mass ratio, the water–binder ratio (volume ratio) of samples with high SS content is larger. In addition, compared with GGBS and FA, SS requires less water for hydration due to less vitreous content; thus, SS improves paste fluidity.

### 3.2. Mechanical Properties

#### 3.2.1. Effects of N_e_ and SS on Compressive Strength of AAM

**Figure 5:**
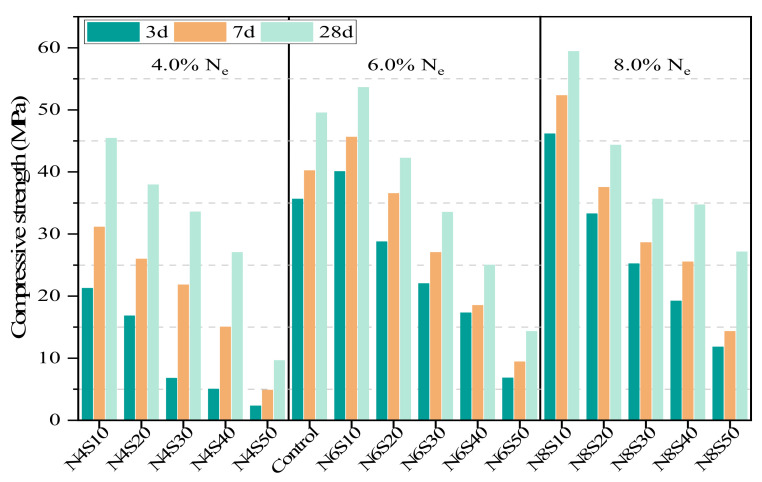
The compressive strength of AAM at different SS contents and N_e_.

Figure 5 shows the compressive strength of the AAM. As can be seen, the compressive strength of the AAM decreases with the increase in SS content when N_e_ is constant. When the content of SS added is 10–20%, the compressive strength at 28 days is close to or even exceeds 40 MPa. When the SS content is increased to 30–40%, the compressive strengths of the AAM with different N_e_ at 28 days were all higher than 30 MPa. When the SS content increase is continued, the paste strength decreases significantly. Moreover, the compressive strength of the AAM increases with the increase in N_e_. Among them, the compressive strength of all samples at 28 days is the highest at 59.4 MPa when N_e_ is 8.0%. The late strength of the samples is significantly higher when N_e_ is 4.0% compared to 6.0% and 8.0% N_e_ samples. Its average compressive strength at 28 days is 55.4% higher than that at 7 days, which is related to the slow reaction process of the paste due to the activation of low alkali concentration [32].

#### 3.2.2. Effects of N_e_ and SS on Flexural Strength of AAM

**Figure 6:**
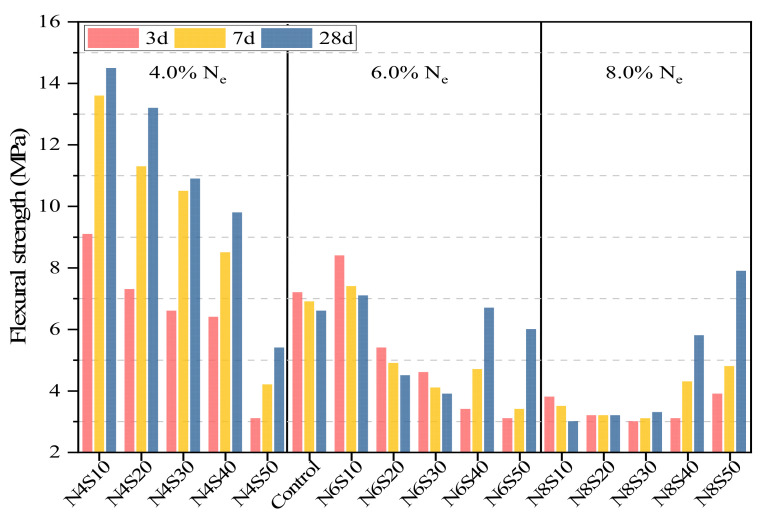
The flexural strength of AAM at different SS contents and N_e_.

Figure 6 shows the flexural strength of the AAM. The flexural strength of the samples containing 4.0% N_e_ decreases with the increase in SS content, but the average value is significantly better than that of 6.0% and 8.0% N_e_ samples. The average flexural strengths are 8.2, 12.45 and 13.85 MPa at 3, 7 and 28 days for 10–20% SS content. However, the 28 days flexural strength was only 5.4 MPa at 50% SS replacement. Interestingly, with increase in the SS content, the flexural strength of the samples with 6.0% and 8.0% N_e_ first decreased and then increased. This is because the violent reaction process leads to the generation of microcracks inside the paste at this N_e_; and the microcracks are connected or expanded with the increase in curing age (see Section 3.4.2), thus reducing the flexural strength. Furthermore, the flexural strength of N6S40, N6S50, N8S40 and N8S50 increases significantly in the later stage of curing. Compared with N8S10, the average flexural strength at 28 days of the 8.0% N_e_ sample mixed with 40–50% SS increased by 128.3%. This is because the microaggregate effect of SS helps prevent the penetration and continuous penetration of internal cracks compared with GGBS and FA (see Section 3.4.2), thereby reducing the adverse effect of alkali on AAM brittleness.

### 3.3. Life Cycle Assessment

#### 3.3.1. GWP Analysis

**Figure 7:**
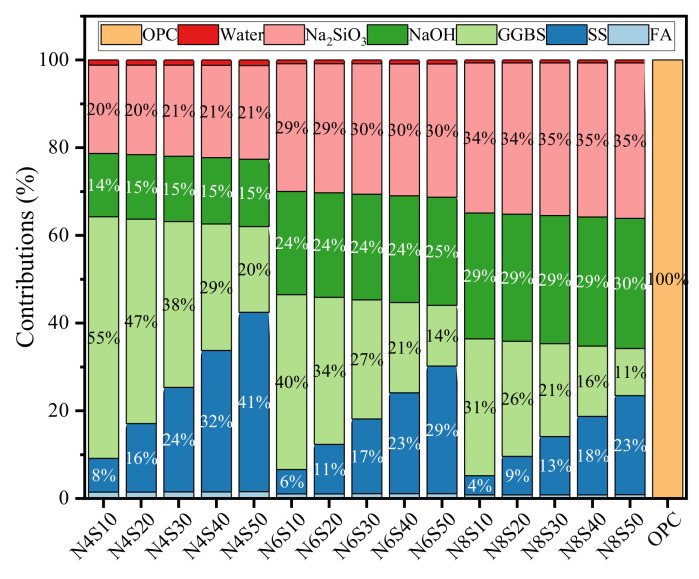
Contribution of raw materials to samples.

**Figure 8:**
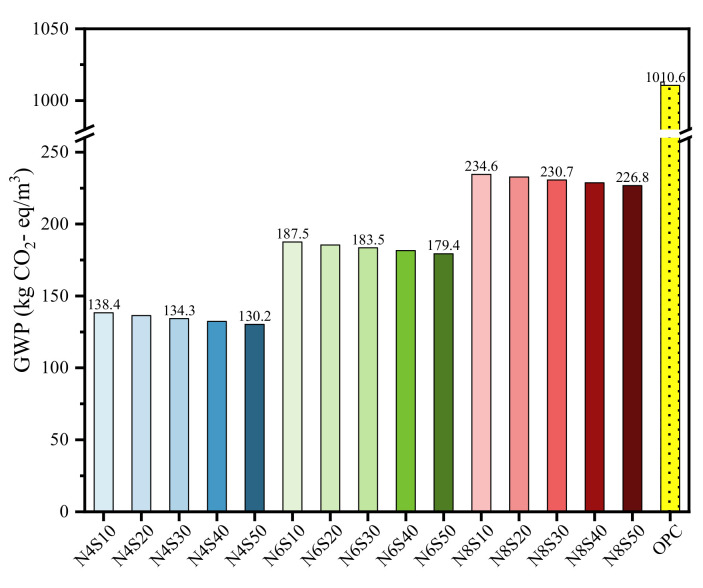
GWP100 of samples.

Table 4 shows the impact of raw materials on GWP100. Based on this, the contribution of each sample to GWP was calculated, as shown in Figure 7. It can be seen that as the N_e_ increases, the GWP contributions of GGBS per cubic meter of the sample significantly decrease, while the contributions of NaOH and Na_2_SiO_3_ are the opposite. Among them, Na_2_SiO_3_ contributed the most to GWP in the samples mixed with 8.0% N_e_ (34–35%), followed by NaOH (28–29%). However, the contribution of OPC in cement to GWP is as high as 99.8%, which is related to the high CO_2_ emissions caused by the decomposition of limestone during the production process [33]. Furthermore, the comparison of the GWP value of each sample is shown in Figure 8. As can be seen, alkali-activated GGBS-SS-FA materials are more excellent in reducing greenhouse gas emissions. OPC has the highest GWP value of 1010.6 kg CO_2_- eq/m^3^. Based on the optimal compressive strength, the GWP value of N8S10 is 76.7% lower than that of cement. The CO_2_ emissions are reduced by 22.97% and 11.41% compared to alkali-activated GGBS [34] and bricks prepared using alkali-activated FA-GGBS [35], respectively. The GWP values of N4S10, N6S10, N6S20 and N8S20 samples that meet the compressive strength requirements of grade 42.5 cement are 86.3%, 81.4%, 81.6% and 76.9% lower than that of cement. The above results indicate that alkali-activated GGBS-SS-FA materials have great potential in reducing carbon emissions.

#### 3.3.2. Impact Potential of Other Environmental Indicators

**Figure 9:**
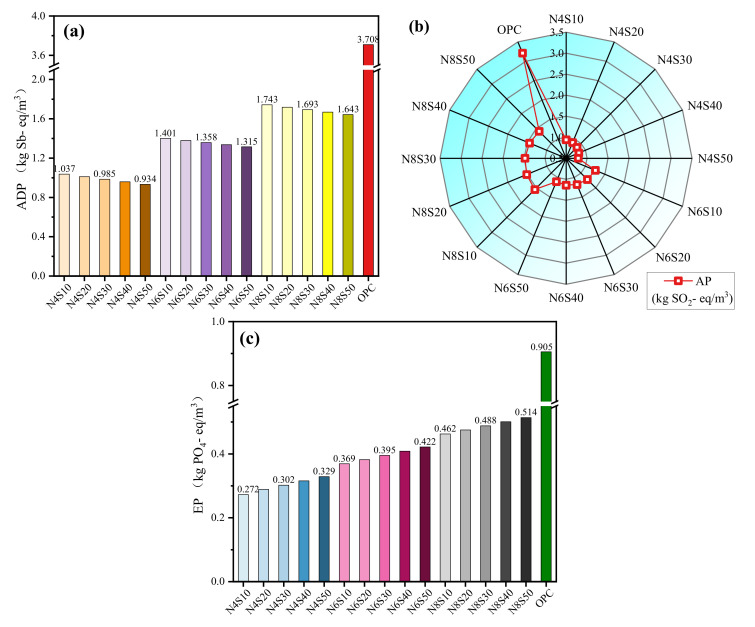
Environmental impacts: (**a**) ADP, (**b**) AP and (**c**) EP.

The impacts of raw materials corresponding to ADP, AP and EP indicators are shown in Table 5. Based on this information, the results of each sample for these impact categories were calculated as shown in Figure 9. It can be seen that as the N_e_ increases, the impacts of each sample on ADP, AP and EP gradually increase; as the SS content increases, ADP and AP gradually decrease and EP gradually increases, but these impacts are smaller than that of cement. ADP is associated with increased consumption of non-renewable resources such as iron, limestone and clay. The ADP values of N4S10, N6S10, N6S20, N8S10 and N8S20 samples are 72.0%, 62.2%, 62.7%, 53.0% and 53.6% lower than that of cement. Compared with ADP and AP, the impact of the mixture on EP is minimal. The average EP of all samples is 3.94 × 10^−1^ kg PO_4_- eq/kg, among which N8S10 has a 48.9% lower value than that of cement. This indicates that the paste prepared in this work can reduce the risk of algae reproduction and deterioration of water quality caused by nutrient enrichment. AP is mainly related to the acidification process caused by gases such as SO_2_ and NO_x_. Compared with cement, the AP values of N4S10, N6S10, N6S20, N8S10 and N8S20 are reduced by 70.6%, 60.9%, 62.1%, 51.6% and 51.7%, respectively. In addition, the compressive strengths of N4S20, N4S30, N6S30, N8S30 and N8S40 are lower than that of cement but higher than 30 MPa; Among them, N4S30 has the lowest compressive strength at 28 days of 33.6 MPa. The ADP, AP and EP values of N4S30 are approximately reduced by 53.09%, 68.4% and 41.92%, respectively, as compared to the bricks prepared from alkali-activated FA-GGBS [35]. Thus, if they are applied to some unreinforced projects, such as pavement bricks, small blocks and other projects that do not have high strength requirements, they can still deliver significant environmental benefits. The above results show that the activated GGBS-SS-FA material can reduce the consumption of raw natural resources and is more conducive to environmental sustainability.

### 3.4. Microscopic Analyses

#### 3.4.1. XRD Analysis

**Figure 10:**
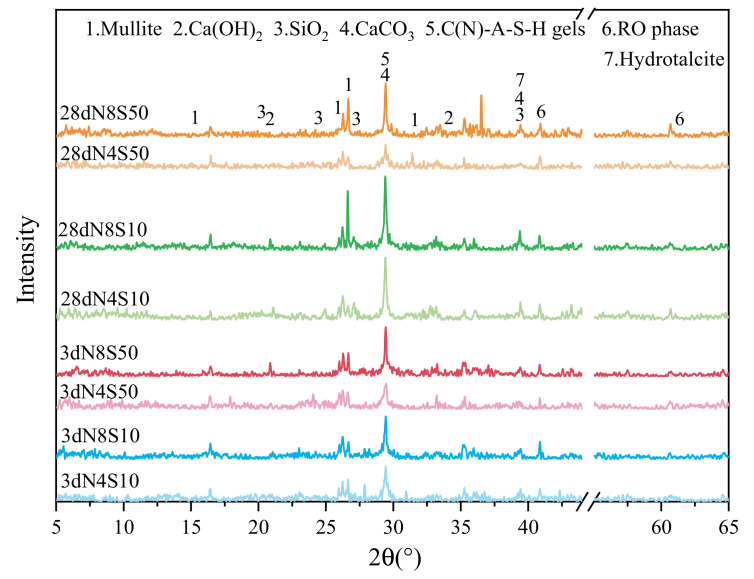
XRD patterns of representative AAM containing SS.

To facilitate the analysis of the effect of SS content and N_e_ on hydration products, the samples from 3 and 28 days with 4.0% and 8.0% N_e_ and SS contents of 10% and 50% were selected for analysis, and their XRD patterns are shown in Figure 10. It can be seen that some mullite and quartz (2θ = 20–25°) originating from unreacted FA particles are observed. All samples exhibit a broad hump in the range of 25–35°, which is the typical XRD peak for an amorphous aluminosilicate framework, indicating the hydration products are mainly C(N)-A-S-H gels [36]. Furthermore, there is a weak CaCO_3_ diffraction peak at 29° (2θ), indicating a small amount of Ca^2+^ in the hardened paste has undergone a carbonization reaction with CO_2_ in air. Weak diffraction peaks of Ca(OH)_2_ are observed at 18° and 24°, which indicates the alkali activator reacted with Ca^2+^ in the system to generate Ca(OH)_2_ and Ca(OH)_2_ participated in the pozzolanic reaction to form gel products. Moreover, no diffraction peaks of ettringite are observed, which is attributed to the low content of sulfur compounds in the raw materials used. There is almost no change in the peak intensity of the RO phase, indicating that the stable RO phase in SS has difficulty in reacting under alkali activation.

A faint hydrotalcite phase is identified in the samples, which is one of the crystalline products of alkali-activated GGBS [37]. Under the same SS content, the main peak intensity of the samples mixed with 8.0% N_e_ was significantly higher than that of samples mixed with 4.0% N_e_ at 3 and 28 days of curing, indicating that the 8.0% N_e_ can promote the depolymerization and polycondensation reaction process of the matrix, thereby generating more C(N)-A-S-H gels. The main peak intensity of the samples doped with 4.0% and 8.0% N_e_ is higher at 28 days than at 3 days of curing, which shows that the activator can continuously release OH^−^ to activate the matrix in the curing age range, thereby allowing the reaction to continue. At the same N_e_ level, the main peak strength of samples at 3 and 28 days of curing decreases as the SS content increases from 10% to 50%, which is attributed to the limited progress of the reaction due to the lower vitreous content of SS compared to that of GGBS.

#### 3.4.2. SEM Analysis

**Figure 11:**
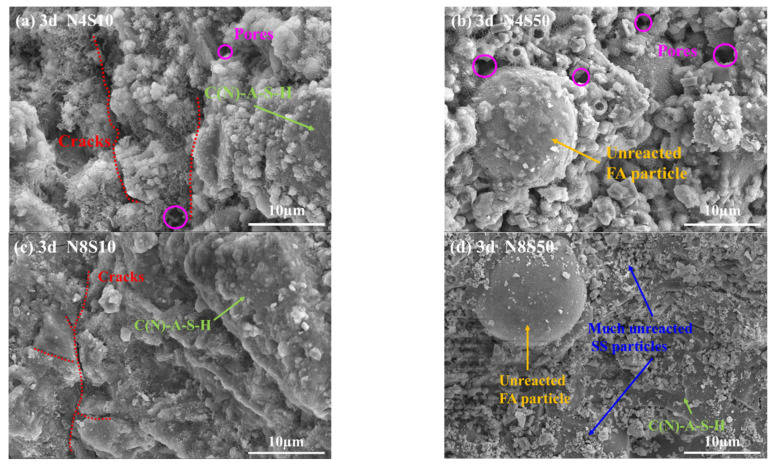
SEM of representative samples at 3 days: (**a**) N4S10, (**b**) N4S50, (**c**) N8S10 and (**d**) N8S50.

SEM pictures of representative samples cured for 3 days are shown in Figure 11. As can be seen, the samples exhibited a typical AAM microstructure consisting of gel products, voids, cracks, residual shell layers and unreacted particles. The gel products of N4S10 and N4S50 at 3 days have relatively weak connections with the residual particles, and the microstructures are relatively disordered with obvious angles, boundaries and pores (Figure 11a,b). As N_e_ increases, the gel products in N8S10 merge with each other and superimpose into a denser whole, but the early violent reaction causes microcracks (Figure 11c); when the SS content is continually increased to 50%, the microcracks in N8S50 are significantly less than that of N8S10, but many unreacted SS particles are attached to the product surface (Figure 11d). Combined with the XRD results, it can be proven that the low glass volume in SS and stable RO phase are reasons for the loose microstructure of N8S50. Moreover, compared with the sample mixed with 4.0% N_e_, the microstructure of the sample mixed with 8.0% N_e_ is smoother and more compact, which again proves that 8.0% N_e_ can activate the matrix in the early stage of the reaction to promote dense-structure formation.

**Figure 12:**
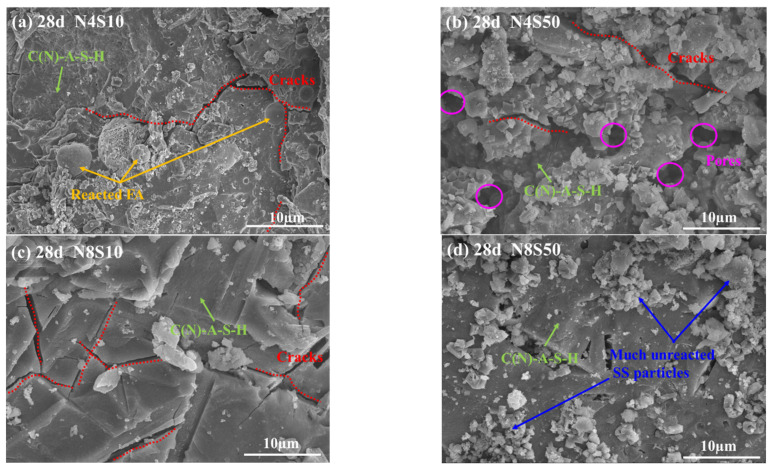
SEM of representative samples at 28 days: (**a**) N4S10, (**b**) N4S50, (**c**) N8S10 and (**d**) N8S50.

The SEM pictures of the representative samples at 28 days are shown in Figure 12. It can be seen that the microstructure of the paste becomes denser and more uniform as the curing age increases. In Figure 12a,b, the shell layer remaining after the FA reaction and the longer cracks developing along the interface are clearly visible in the matrix. Notably, most of the SS particles in N4S10 and N8S10 have reacted or been embedded in the gel products by physical filling at 28 days compared to 3 days. Combined with the compressive strength results, it can be shown that incorporating 10% SS into the system can give full play to its microaggregate effect and help improve strength development to a certain extent. There are microcracks in N8S10, and the cracks gradually expand with the increase in the curing age (Figure 12c), which is an important reason for the decrease in flexural strength of the samples with 6.0% and 8.0% N_e_. Compared to N8S10, few microcracks are observed in N8S50 at 28 days (Figure 12d), but there are significantly more unreacted SS particles. Consistent with the mechanical property results, SS can inhibit crack generation and thus improve paste brittleness, but if its content is higher than 10%, it will lead to a decrease in the mechanical properties due to the poor activity of the system.

#### 3.4.3. NMR Analysis

**Figure 13:**
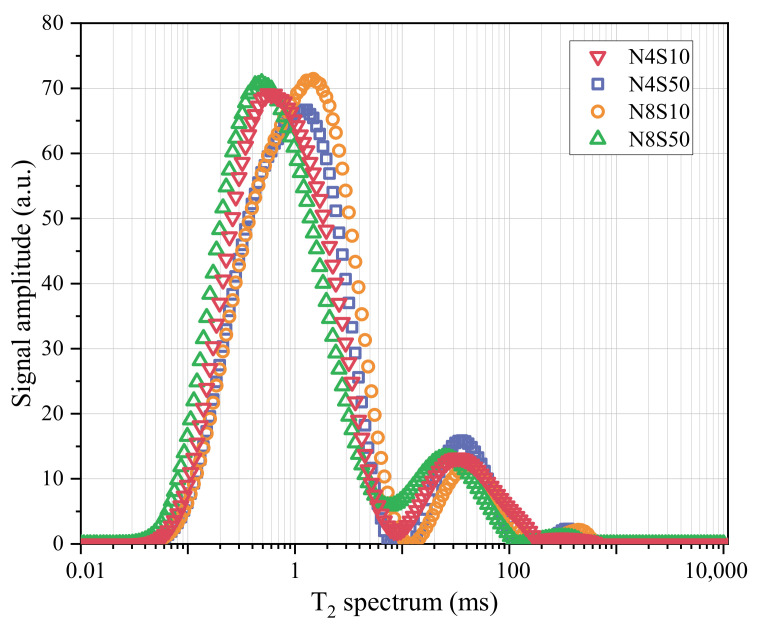
T_2_ spectrum of representative samples at 28 days.

The T_2_ spectra of the representative samples at 28 days are shown in Figure 13. It can be seen that the T_2_ spectra of the samples exhibit a standard three-peak pattern, with two minor peaks (peaks 2 and 3) and one primary peak (peak 1). The larger the pore size of the porous medium, the longer the T_2_ relaxation time; thus, peaks 1, 2 and 3 can represent small, medium and large pores in the sample [38]. The signal intensity and area of the primary peak are much higher than that of the secondary peak, indicating that the pores in the sample are mainly composed of small pores.

When a sample is totally saturated with water, the T_2_ spectral area can be regarded as its porosity [39]. Thus, the area and proportion of each peak were calculated, as shown in Table 6. Under the same SS content, the T_2_ spectral area of N8S10 decreased by 4.84% compared with N4S10 and N8S50 decreased by 4.02% compared with N4S50 after 28 d of curing. This indicates that 8.0% N_e_ can promote the paste reaction and produce more reaction products to refine the pore structure. At the same N_e_ content, the T_2_ spectral area of N8S50 is increased by 6.94% compared to N8S10, and N4S50 is increased by 6.02% compared to N4S10. This again shows that too much SS content in the system is prone to insufficient reaction, which leads to a loose and porous structure. Notably, the large pore ratio (peak 3) of N8S10 is higher than that of N8S50, which is consistent with the SEM observation results. The violent geopolymerization reaction leads to microcrack generation in the paste mixed with 8.0% N_e_, which increases the large pore proportion in N8S10; the proportion of large pores decreases as the content of SS increases, which once again proves that SS can play a role in the system as a physical filling effect.

#### 3.4.4. Mechanism Analysis

**Figure 14:**
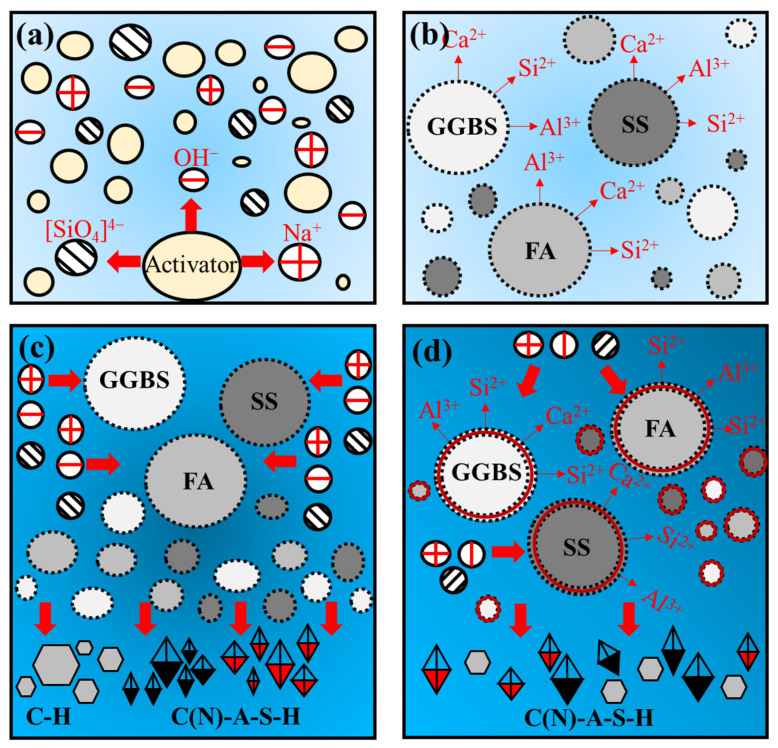
Schematic diagram of the mechanism of AAM: (**a**) activator hydrolysis, (**b**) dissolution process, (**c**) polycondensation process and (**d**) dissolution and re-polycondensation process.

Based on the former discussion, the reaction mechanism of the AAM containing SS is summarized. Figure 14 shows the particle surface dissolution and reaction process of the raw materials, and the reaction equations are shown in Equations (3)–(8).

Firstly, the alkali activator is dissolved in water to release hydroxide and silicate ions (Figure 14a), increasing the pH value of the solution. Under an alkaline environment, the surface layer of the precursors gradually disintegrates. The Ca-O, Si-O-Si and Al-O-Al bonds break and react to form silicate monomers and aluminum monomers (Figure 14b; Equations (3)–(5)) [40,41]. Subsequently, silicate monomers and aluminum monomers react with calcium or sodium ions to form C(N)-A-S-H gel; calcium ions react with OH^−^ to form C-H gel (Figure 14c; Equations (6)–(8)) [42,43]. Furthermore, the disruption of the calcium, silicon and aluminum phases provides a gateway for the alkali activators to enter the interior of the bonded particles, whereby the bonded particles are continuously activated and reaction products are generated (Figure 14d). As the reaction proceeds, the gel monomer particles cross-link and fuse to form a dense network structure and intertwine with unreacted particles and form a skeletal structure, which improves the paste strength.

SiO_2_ + OH^−^ + H_2_O → [H_3_SiO_4_]^−^ (3)

AlO_2_^−^ + OH^−^ + H_2_O → [H_3_AlO_4_]^2−^ (4)

AlO_2_^−^ + OH^−^ + H_2_O → [Al(OH)_6_]^3−^ (5)

Ca^2+^ + 2[H_3_AlO_4_]^2−^ + 2[H_3_SiO_4_]^−^ → CaAl_2_Si_2_O_8_·4H_2_O + 4OH^−^ (6)

2Na^+^ + 4[H_3_AlO_4_]^2−^ + 4[H_3_SiO_4_]^−^ → 2NaAl_2_Si_2_O_8_·4H_2_O + 8OH^−^ (7)

2Ca^2+^ + OH^−^ → Ca(OH)_2_ (8)

## 4. Conclusions

A green and high-strength AAM containing SS was prepared in this work. The effects of alkali equivalent and SS content on the properties and microstructure development of the AAM were investigated. Based on the experimental results, the following conclusions can be drawn:

(1) Although the increase in GGBS content and alkali equivalent speeds up the early reaction rate of the paste, the rapid generation of reaction products reduces fluidity and setting time. The incorporation of SS can improve the fluidity and setting time of the paste to a certain extent, which is mainly related to the slowdown of the paste reaction rate caused by the reduction in the vitreous content and hydration water demand in the system.
(2) Thanks to the geopolymerization reaction and physical filling effect of SS, the highest compressive strength of the paste is obtained when the SS content is 10%. In addition, the significant increase in the compressive strength of the paste with the increase in the alkali equivalent from 4.0% to 8.0% is related to the accelerated reaction process, the increase in hydration products and refinement of the pores.
(3) With the gradual increase in the alkali equivalent, the microcracks in the hardened paste caused by thermal stress gradually increase and the cracks gradually expand with the increase in the age of curing, which is the main reason for the decrease in the flexural strength. The microaggregate effect exerted by SS is significant when the content of SS is 40% and 50%, which blocks the developing cracks and improves the paste brittleness.
(4) The paste reaction product mainly consists of C(N)-A-S-H, hydrotalcite, Ca(OH)_2_ and CaCO_3_. As the alkali equivalent increases from 4.0% to 8.0%, the gel products gradually stack and cement together with unreacted particles, which improves the microstructure compactness. However, when the SS content increases from 10% to 50%, the unreacted SS gradually increases and adheres to the surface of the reaction product, which results in a loose and rough microstructure.
(5) The life cycle assessment shows that the average GWP, ADP, AP and EP values of all the samples prepared in this work that meet the compressive strength requirements of grade 42.5 cement are reduced by 80.5%, 60.7%, 59.3% and 56.6% compared to that of cement. The reuse of solid waste such as steel slag and fly ash reduces original resource consumption and CO_2_ emissions, which has a guiding significance for the recycling and low-carbonization of solid waste in the engineering field.

## Figures and Tables

**Table 1:** Chemical compositions of industrial solid wastes (w/%).

Composition	CaO	SiO_2_	Al_2_O_3_	Fe_2_O_3_	SO_3_	MgO	K_2_O	Na_2_O	Other	LOI
FA	2.79	51.51	37.53	3.15	0.99	0.52	0.99	1.49	0.38	0.65
GGBS	39.46	31.17	15.01	0.28	0.59	8.91	0.28	0.46	2.53	1.31
SS	32.57	21.67	7.35	21.85	0.79	6.46	0.55	0.49	6.08	2.19

**Table 2:** Chemical and physical properties of sodium silicate.

Items	Baume	Na_2_O (%)	SiO_2_ (%)	H_2_O (%)	Density (g/mL)	Modulus
Results	40	8.3	26.5	65.2	1.371	3.2

Baume: another method used to indicate the concentration of a solution in production process.

**Table 3:** Mix proportions of AAMs.

Sample ID	Mix Proportion of Binder by Weight (g)			N_e_ (External Addition)	Water–Binder Ratio
	SS	GGBS	FA		
Control		700	300	6.0%	0.45
N4S10	100	600		4.0% (235.71 g modified Na_2_SiO_3_)	
N4S20	200	500			
N4S30	300	400			
N4S40	400	300			
N4S50	500	200			
N6S10	100	600		6.0% (353.565 g modified Na_2_SiO_3_)	
N6S20	200	500			
N6S30	300	400			
N6S40	400	300			
N6S50	500	200			
N8S10	100	600		8.0% (471.42 g modified Na_2_SiO_3_)	
N8S20	200	500			
N8S30	300	400			
N8S40	400	300			
N8S50	500	200			

**Table 4:** Impact of raw materials evaluated (GWP100).

Raw Materials	FA	SS	GGBS	NaOH	Na_2_SiO_3_	Water	OPC
GWP (kg CO_2_- eq/kg)	4.00 × 10^−2^	6.30 × 10^−2^	7.51 × 10^−2^	1.48 × 10^0^	8.23 × 10^−1^	2.14 × 10^−3^	8.49 × 10^−1^

**Table 5:** Impact of raw materials in environmental impact categories.

Raw Materials	Abiotic Resource Depletion Potential (ADP)	Acidification Potential (AP)	Eutrophication Potential (EP)
	kg Sb- eq/kg	kg SO_2_- eq/kg	kg PO_4_- eq/kg
FA	3.29 × 10^−10^	7.26 × 10^−6^	1.05 × 10^−6^
SS	4.47 × 10^−4^	3.32 × 10^−4^	2.17 × 10^−4^
GGBS	6.00 × 10^−4^	5.58 × 10^−4^	1.38 × 10^−4^
NaOH	1.04 × 10^−2^	6.87 × 10^−3^	2.81 × 10^−3^
Na_2_SiO_3_	6.26 × 10^−3^	6.53 × 10^−3^	1.68 × 10^−3^
Water	1.34 × 10^−6^	1.29 × 10^−6^	2.28 × 10^−7^
OPC	2.15 × 10^−3^	1.86 × 10^−3^	5.25 × 10^−4^

**Table 6:** NMR spectral area and proportion.

Samples	T_2_ Spectral Area	Peak 1		Peak 2		Peak 3	
		Area	Proportion	Area	Proportion	Area	Proportion
N4S10	3012.396	2666.22	88.508	333.894	11.084	12.282	0.408
N4S50	3193.792	2920.092	91.43	235.942	7.388	37.758	1.182
N8S10	2866.567	2558.843	89.265	292.971	10.22	14.752	0.515
N8S50	3065.423	2724.849	88.89	327.059	10.669	13.515	0.441

## Data Availability

Dates are contained within the article.
